# *Mycobacterium tuberculosis* β-lactamase variant reduces sensitivity to ampicillin/avibactam in a zebrafish-*Mycobacterium marinum* model of tuberculosis

**DOI:** 10.1038/s41598-023-42152-8

**Published:** 2023-09-16

**Authors:** Ilona van Alen, Mayra A. Aguirre García, Janneke J. Maaskant, Coenraad P. Kuijl, Wilbert Bitter, Annemarie H. Meijer, Marcellus Ubbink

**Affiliations:** 1 Leiden Institute of Chemistry, Leiden University, Einsteinweg 55, 2333CC Leiden, The Netherlands; 2 Institute of Biology Leiden, Leiden University, Einsteinweg 55, 2333CC Leiden, The Netherlands; 3 Department of Medical Microbiology and Infection Control, Amsterdam UMC, Location VUmc, De Boelelaan 1108, 1081 HZ Amsterdam, The Netherlands; 4 Section of Molecular Microbiology, Amsterdam Institute of Molecular and Life Sciences, Vrije Universiteit Amsterdam, De Boelelaan 1108, 1081 HZ Amsterdam, The Netherlands

**Keywords:** Molecular evolution, Infection, Zebrafish, Biochemistry, Enzymes, Proteins, Bacteria

## Abstract

The β-lactamase of *Mycobacterium tuberculosis*, BlaC, hydrolyzes β-lactam antibiotics, hindering the use of these antibiotics for the treatment of tuberculosis. Inhibitors, such as avibactam, can reversibly inhibit the enzyme, allowing for the development of combination therapies using both antibiotic and inhibitor. However, laboratory evolution studies using *Escherichia coli* resulted in the discovery of single amino acid variants of BlaC that reduce the sensitivity for inhibitors or show higher catalytic efficiency against antibiotics. Here, we tested these BlaC variants under more physiological conditions using the *M. marinum* infection model of zebrafish, which recapitulates hallmark features of tuberculosis, including the intracellular persistence of mycobacteria in macrophages and the induction of granuloma formation. To this end, the *M. tuberculosis blaC* gene was integrated into the chromosome of a *blaC* frameshift mutant of *M. marinum.* Subsequently, the resulting strains were used to infect zebrafish embryos in order to test the combinatorial effect of ampicillin and avibactam. The results show that embryos infected with an *M. marinum* strain producing BlaC show lower infection levels after treatment than untreated embryos. Additionally, BlaC K234R showed higher infection levels after treatment than those infected with bacteria producing the wild-type enzyme, demonstrating that the zebrafish host is less sensitive to the combinatorial therapy of β-lactam antibiotic and inhibitor. These findings are of interest for future development of combination therapies to treat tuberculosis.

## Introduction

One-quarter of the world’s human population is estimated to be latently infected with tuberculosis (TB) and TB is the leading cause of death by bacterial infection in 2020^1^. *Mycobacterium tuberculosis* (Mtb), the pathogen that causes TB, can be transmitted between hosts in aerosol particles and parasitizes macrophages in the lungs of the patient^2^. Mtb expresses the *blaC* gene, coding for a class A extended-spectrum β-lactamase, BlaC, that can hydrolyze β-lactam antibiotics, such as ampicillin and early generations of cephalosporins^3,4^. The presence of this protein prohibits the use of β-lactam antibiotics for the treatment of TB. However, the discovery and development of β-lactamase inhibitors opens up possibilities for combination therapies. Clavulanic acid is a naturally occurring β-lactam that is produced by *Streptomyces clavuligerus* and has FDA approval to be used in combination with several antibiotics for the treatment of bacterial infections in both humans and domestic animals^5,6^. The World Health Organization has classified amoxicillin-clavulanic acid in combination with meropenem as a possible add-on-agent in the treatment of patients with multi-drug-resistant or rifampicin-resistant TB, but the evidence for successful outcomes is sparse^7–11^. Sulbactam and avibactam are both synthetic inhibitors, the former structurally similar to clavulanic acid, while the latter is a diazabicyclooctane. Sulbactam is in clinical use in combination with ampicillin, cefoperazone, or durlobactam, and avibactam is combined with ceftazidime^12–14^. Both inhibitors are used to treat infection caused by Gram-negative bacteria, and sulbactam can also be used for Gram-positive organisms. Neither is currently used to treat TB. In vitro data show that these inhibitors inhibit BlaC by covalently binding to the catalytic residue Ser70^15^.

Previous experiments using *Escherichia coli* as a host have shown that BlaC can be inhibited by β-lactamase inhibitors^16–18^. Single amino acid mutations have been identified that improve catalytic efficiency against ampicillin or reduce sensitivity for inhibitors^16,18–22^. It is currently unknown whether these phenotypes of BlaC variants translate to more physiological conditions*.* Therefore, we aimed to test the effect of amino acid mutations using the zebrafish-*Mycobacterium marinum* infection model.

Zebrafish (*Danio rerio*) are widely used as a model system of tuberculosis for both active and latent disease^23–27^. *M. marinum* (Mmar) is a natural pathogen of zebrafish that shares 3000 orthologs with Mtb, including crucial virulence factors, with an average sequence similarity of 85%^28^. After the infection of zebrafish by Mmar, the bacteria are phagocytosed by macrophages, which subsequently invade tissues and initiate the formation of granulomas^29^. These are collections of infected and uninfected macrophages surrounded by epithelial cells and other immune cells and are similar to granulomas found in the lungs of human patients infected with Mtb^30–34^. The early stages of granuloma formation can be studied in zebrafish embryos, which are optically transparent, allowing for easy and non-invasive visualization of cell tissues or bacteria using fluorescent labels. Mmar infection studies in zebrafish embryos have contributed key insights into the function of granulomas and the role of different immune response genes in host resistance^26,34,35^. Furthermore, the zebrafish embryo model has successfully been used to screen for novel anti-infectious treatments^36–38^.

Here, we aimed to establish whether the properties of BlaC variants result in changed infection patterns in the zebrafish TB model. A *blaC* knockout strain of Mmar was created and Mtb *blaC* variants were introduced for chromosomal expression. Growth on plates shows the same trends as the data obtained previously with an expression system in *E. coli*, indicating that this organism yields representative data. Furthermore, the zebrafish model system shows that inhibition effects of wild-type BlaC and BlaC K234R observed in Mmar and *E. coli* lead to the predicted changes in infection levels, demonstrating that ampicillin/avibactam is effective as treatment of Mmar infection in zebrafish.

## Results

### Mmar expressing BlaC variants shows reduced sensitively to β-lactam inhibitors

**Figure 1:**
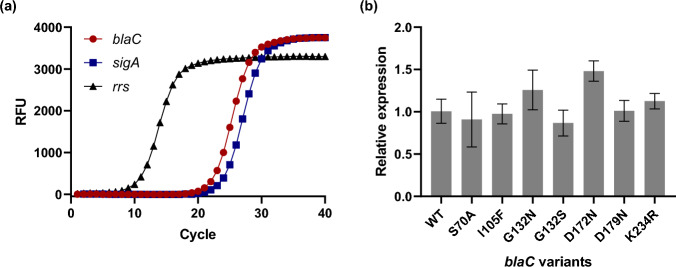
Expression of Mtb *blaC* in *Mmar.* (**a**) Amplification of *blaC* and housekeeping genes *sigA* and *rrs* during one-step RT-qPCR using RNA isolated from Mmar expressing wild-type Mtb *blaC* as template. (**b**) Relative expression of *blaC* variants as determined by RT-qPCR. Data represent three biological replicates, each with two technical replicates, and the *blaC* transcripts were normalized to *sigA*. The error bars represent one standard deviation. One-way ANOVA with Dunnett’s multiple comparison test indicates a significant difference between *blaC* WT and *blaC* D172N (*p* < 0.05).

We first aimed to test the effect of single amino acid mutations and compare them with other studies on Mtb BlaC. Mmar BlaC and Mtb BlaC share only 70% of their amino acid sequence, necessitating the replacement of Mmar *blaC* by the homologous Mtb gene. *Streptococcus thermophilus* CRISPR1-Cas9 was used to produce a frameshift in the Mmar *blaC* gene^39^, resulting in a stop codon after Ser31 (Ambler numbering)^40^. A mycobacterial integration vector was used to introduce the Mtb *blaC* sequence coding for the mature protein, preceded by the promotor region and signal sequence of Mmar *blaC*, into the chromosome of Mmar (Fig. S1). The transcript level of Mtb *blaC* was determined by reverse transcription-quantitative PCR (RT-qPCR) and found to be comparable to that of the housekeeping gene *sigA*. Only small differences were observed between the different *blaC* variants used in this study (Fig. 1).

**Figure 2:**
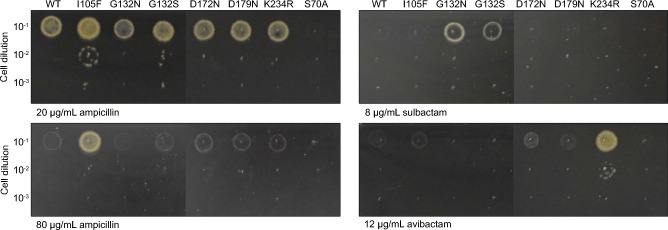
Activity of Mtb BlaC mutants produced in Mmar*.* Cultures of Mmar chromosomally expressing wild-type BlaC or variants S70A (negative control), I105F, G132N, G132S, D172N, D179N, and K234R were incubated for 8 days at 30 °C on plates containing 20 or 80 μg mL^−1^ ampicillin, or 8 μg mL^−1^ sulbactam or 12 μg mL^−1^ avibactam in the presence of 15 μg mL^−1^ ampicillin. The complete plates are shown in Figs. S2–S4.

The strains were tested for their ability to grow in the presence of either ampicillin or carbenicillin, or a combination of ampicillin and inhibitor (Figs. 2, S2, and S3). The strain carrying the wild-type Mtb *blaC* is able to grow on a plate with these antibiotics. Mutation S70A results in a catalytically inactive BlaC, as it prohibits the formation of the acyl-enzyme. Mmar expressing this variant does not grow in the presence of ampicillin or carbenicillin, serving as a negative control. Ile105 has been named the gatekeeper residue^21^, and is present in the loop that restricts access to the active site. A Phe in position 105 improves the catalytic efficiency of the enzyme and allows for hydrolysis of ampicillin in the presence of clavulanic acid^21^. Production of Mtb BlaC I105F allows Mmar to grow on 80 μg mL^−1^ ampicillin, whereas Mmar expressing the wild-type Mtb *blaC* or other variants grow up to 40 μg mL^−1^ ampicillin. Position 132 is generally occupied by Asn in class A β-lactamases, yet BlaC has Gly in this position. Mutation G132N restores the canonical motif, which was reported to result in reduced sensitivity for both clavulanic acid and avibactam^19,20,41^, while G132S was found in laboratory evolution experiments to confer reduced sensitivity for sulbactam and increased sensitivity for avibactam^16^. When expressed in Mmar, the mutation of residue 132 to either Asn or Ser was observed to confer reduced sensitivity for sulbactam. BlaC K234R was previously found to reduce sensitivity for clavulanic acid^18,20^ and the K234R mutation is known to confer reduced sensitivity for avibactam to the β-lactamase KPC-2^42^. In Mmar, Mtb BlaC K234R also confers reduced sensitivity for avibactam. The other mutations tested, D172N and D179N, decreased sensitivity for sulbactam and avibactam, respectively, when produced in *E. coli*^16,22,43^*.* This is likely due to increased protein stability, and no such decrease was observed in Mmar. The addition of clavulanic acid to the plates containing ampicillin showed no effect on bacterial growth for the concentrations tested (Fig. S4), probably caused by the instability of clavulanic acid at 30 °C in combination with the long growth time in these experiments (8 days)^44,45^*.* In conclusion, most of the mutation effects previously observed with *E. coli* as a host translate to Mtb *blaC* expressed in Mmar*.*

### Inhibition of BlaC in zebrafish embryos infected with Mmar

To test whether ampicillin, clavulanic acid, sulbactam, and avibactam could be used to treat zebrafish for mycobacterial infections, embryotoxicity tests were performed by injecting high concentrations of the compounds into the blood island of the embryos. After 4 days, no developmental abnormalities were observed for any groups and more than 80% of the injected larvae survived (Fig. S5a–d), similar to the non-injected controls.

**Figure 3:**
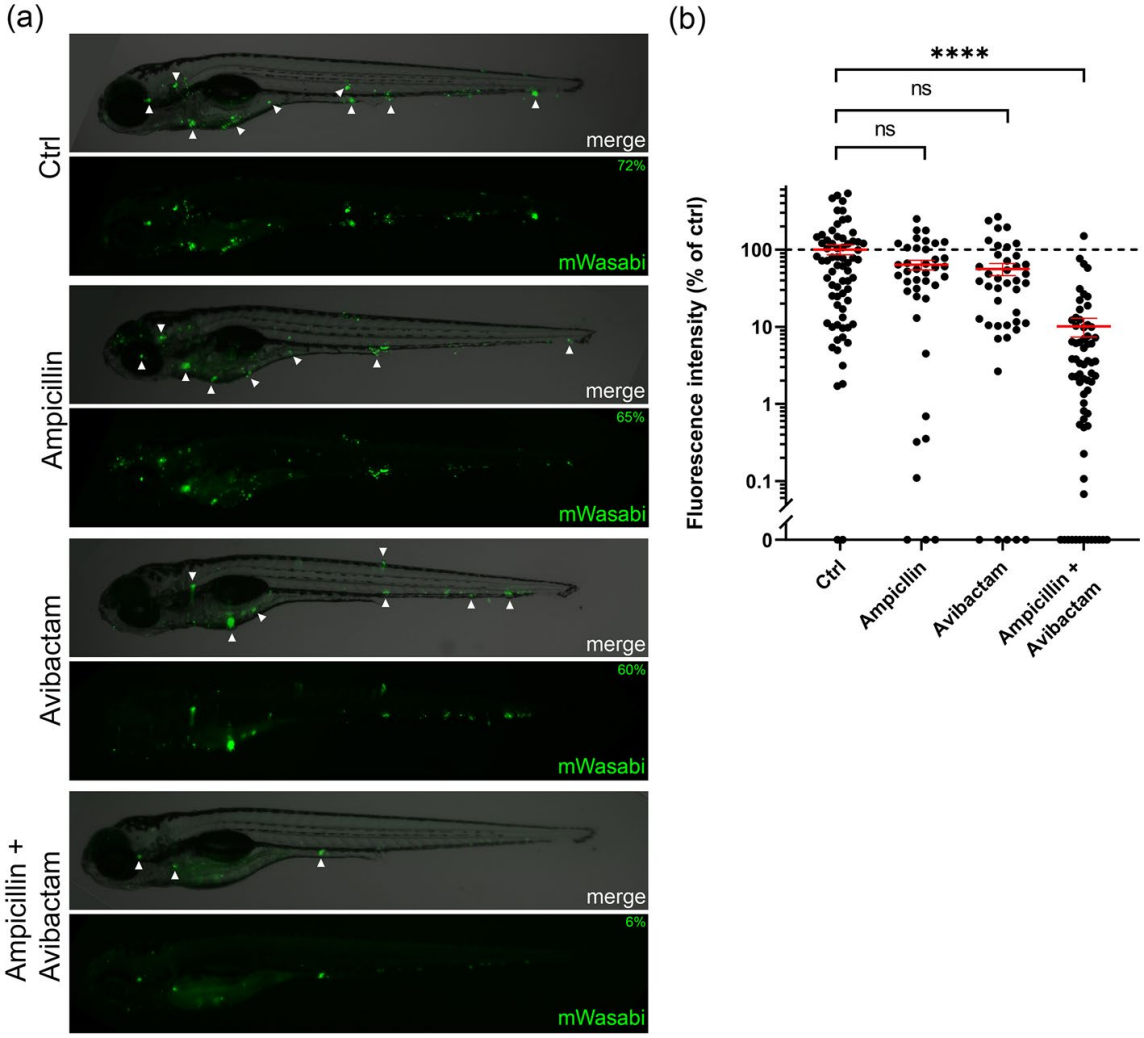
Effect of combination treatment on zebrafish embryos infected with mWasabi-labelled Mmar producing Mtb BlaC at 4 dpi. (**a**) Representative larvae showing systemic infection from the control group and treated with ampicillin, avibactam, or both. Arrows indicate collections of bacteria indicative of granuloma formation, and percentages indicate the percentage of the mean of the control. The control represents a picture of a larva just below the average value of 100% intensity (72%). (**b**) Bacterial load of Mmar producing wild-type Mtb BlaC as represented by fluorescence intensity after being given the indicated treatment 1 dpi. Each dot represents a larva. Larvae were injected with 1 nL of 29 mg mL^−1^ ampicillin in PBS (estimated concentration 100 μg mL^−1^ in the embryo, n = 39), 65 mg mL^−1^ avibactam in PBS (225 μg mL^−1^, n = 43), ampicillin and avibactam in PBS (same concentrations, n = 67), or PBS only (ctrl, n = 69). Data for the groups treated with both ampicillin and avibactam or the control were accumulated in three, and the groups treated with only ampicillin or avibactam in two independent experiments. Data were normalized by setting the mean of the control to 100%. Error bars represent the mean and standard error. Mood’s median test with Holm-Bonferroni post hoc test for multiple comparisons was used to compare groups with the control: ns = not significant; **** = *p* < 0.0001.

To test the effect of β-lactam antibiotics and inhibitors on Mtb BlaC activity in zebrafish embryos, ampicillin and avibactam were selected as treatment as they result in a clear difference in phenotype for BlaC variants on plate (Fig. 2). Embryos were injected in the blood island with Mmar expressing Mtb *blaC* and producing the fluorescent protein mWasabi around 30 h post fertilization. Embryos showing systemic infections were treated 24 h after infection by injection in the Duct of Cuvier with PBS, ampicillin, avibactam, or both and the bacterial load was determined 4 days after infection. Embryos injected with PBS showed clear signs of infection, with the formation of the typical granuloma-like aggregates of infected cells, as did those treated with ampicillin or avibactam (Figs. 3 and S6a). Zebrafish embryos treated with both ampicillin and avibactam showed lower fluorescence intensity than untreated larvae, indicating a reduced bacterial load. Interestingly, the same effect was observed for larvae infected with Mmar harboring native BlaC (Figs. S5e and S6c). Injection of ampicillin or avibactam alone had no significant effect on infection. These results show that ampicillin/avibactam combination therapy is effective in the zebrafish TB model.

### Mutation K234R reduces sensitivity to ampicillin/avibactam in zebrafish

**Figure 4:**
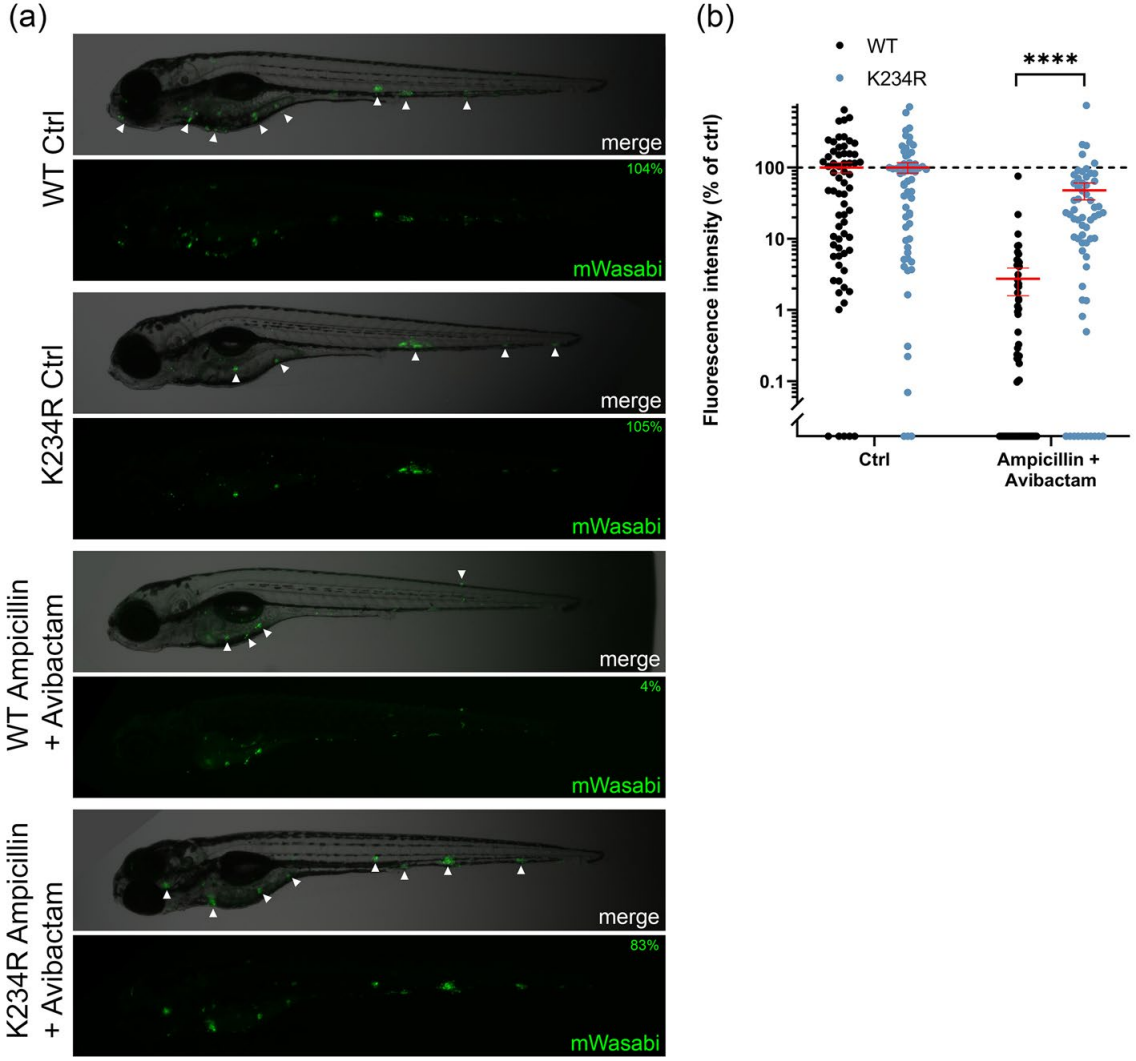
Effect of combination treatment on zebrafish embryos infected with mWasabi-labelled Mmar producing Mtb BlaC variants at 4 dpi. (**a**) Representative larvae showing systemic infection from the control group and treated with ampicillin, avibactam, or both. Arrows indicate collections of bacteria indicative of granuloma formation, and percentages indicate the percentage of the mean of the control. (**b**) Bacterial load of Mmar producing either wild-type (black dots) or K234R Mtb BlaC (blue dots) as represented by fluorescence intensity after being given the indicated treatment 1 dpi. Each dot represents a larva. Larvae were injected with 1 nL of 29 mg mL^−1^ ampicillin and 22 mg mL^−1^ avibactam in PBS (estimated concentration 100 μg mL^−1^ ampicillin and 75 μg mL^−1^ avibactam in the embryo, n = 69 for WT and n = 62 for K234R) or PBS only (ctrl, n = 66 for WT and n = 63 for K234R). Data were accumulated in three independent experiments and normalized by setting the mean of the control to 100%. Error bars represent the mean and standard error. Mood’s median test was used to compare treated groups: **** = *p* < 0.0001.

To check whether the properties of BlaC variants could influence Mmar pathogenesis in zebrafish, we used the K234R mutation that moderately reduces the sensitivity for avibactam, by eightfold and tenfold in growth assays for KPC-2 in *E. coli* and BlaC in Mmar, respectively^42^. Zebrafish embryos were infected with Mmar strains producing either wild-type BlaC or BlaC K234R and those with systemic infections were injected with a combination of ampicillin and avibactam 24 h after infection. Infection levels in the control group were not influenced by the mutation, but a clear difference was observed for the treatment group (Figs. 4 and S6b). In the presence of ampicillin/avibactam combination therapy, infection levels in larvae infected with Mmar producing BlaC K234R were significantly higher than the infection levels for wild-type BlaC. Therefore, we conclude that the K234R mutation reduces sensitivity to ampicillin/avibactam in the zebrafish Mtb BlaC model.

## Discussion

In vivo experiments testing inhibition of Mtb BlaC are generally performed using *E. coli.* While this method is efficient, *E. coli* bacteria differ substantially from mycobacteria in terms of accessory proteins, cell physiology and cell wall composition, posing the question whether results obtained in *E. coli* translate to Mtb. Furthermore, it is not clear whether the observed phenotypes affect the pathogenic behavior of mycobacteria in vivo. Here, we aimed to test inhibition of wild-type BlaC and the effect of previously documented mutations in Mmar, using both a growth assay on plate and an infection assay in zebrafish embryos that is used as a model for TB in humans. Six mutants were compared to both wild-type BlaC and the catalytically inactive mutant S70A. It was found that the effects on enzyme activity and inhibition were similar to the effects previously found using *E. coli*^16,21,22^*.* Only the marginal increases in antibiotic resistance of cells that was ascribed to enhanced enzyme stability of the variants could not be observed in Mmar.

Interestingly, the concentrations for antibiotics and inhibitors at which Mmar growth is inhibited are comparable to concentrations used in growth assays using *E. coli*^16,21,43^, while the permeability of the mycobacterial outer membrane has been reported to be 1000 times lower than for *E. coli*^46,47^*.* Nevertheless, it was reported before that concentrations of both β-lactam and other antibiotics used for the selection of bacteria during cloning are similar for *E. coli* and Mmar^48^*.* Addition of detergent, such as Tween 80, which is added to 7H9 medium to prevent aggregation, probably disorganizes the cell wall, making it more permeable^49,50^*.* While Tween 80 was present in the liquid cultures used to grow the Mmar bacteria, and therefore present in the drops placed on the plates, it was absent from the plates themselves during the growth assays.

Zebrafish embryos and larvae are often used for high-throughput screens in drug research^51–53^ Potential drugs are added to the egg water rather than injected in the embryo, and the drugs should diffuse through the embryonic skin. However, not all small molecules are readily taken up and this mechanism differs from mammalian uptake^53–56^*.* Here, we injected the drugs into the Duct of Cuvier to precisely control concentrations ensuring full bioavailability for all individual larvae. Our results demonstrate that introduction of the Mtb BlaC K234R variant into Mmar reduces sensitivity to ampicillin/avibactam in the zebrafish TB model. It will be of interest to establish whether the results are also of relevance to lower the bacterial burden of Mtb in human cells.

Previous studies tested the inhibition of β-lactamases in zebrafish used either *Mycobacterium abscessus* or *Staphylococcus aureus* expressing their native β-lactamases^57–59^*.* Treatment with amoxicillin and avibactam decreased both the development of abscesses and the overall mortality when compared to fish treated with only amoxicillin 13 days post-infection with *M. abscessus* expressing Bla_Mab_^57^*.* Combining avibactam with imipenem had a similar effect^58^.

## Conclusion

In conclusion, the study shows that results obtained with mutants in the rather reductionistic and simple one gene—one phenotype system of *blaC* expression in *E. coli* are representative of the phenotype of these mutants in a mycobacterium and in the zebrafish TB model. The model offers the possibility to test for the effects of antibiotic/inhibitor combinations, also together with other therapies, in an efficient way and to determine the evolvability of resistance due to mutations in BlaC.

### Materials and methods

#### Bacterial strains, media, and zebrafish handling

All mycobacterium strains were derived from Mmar M and cultured in Middlebrook 7H9 medium, supplemented with 10% ADC and 0.05% Tween 80 or plated on Middlebrook 7H10 agar containing 10% ADC/OADC and 0.5% glycerine^60^*.* Antibiotics were added when appropriate at concentrations of 50 μg mL^−1^ kanamycin, 30 μg mL^−1^ streptomycin, or 50 μg mL^−1^ hygromycin. Cultures and plates were incubated at 30 °C. *E. coli* KA797 cells were used to clone and generate plasmids, and incubated in LB medium or on agar plates with appropriate antibiotics at 37 °C^61^. Zebrafish (*D. rerio*) of wild-type line AB/TL (a cross between AB and Tuebingen Longfin) were maintained and handled according to the guidelines from the Zebrafish Model Organism Database (http://zfin.org) and in compliance with international guidelines specified by the EU Animal Protective Directive 2010/63/EU. They were exposed to a cycle of 14 h light and 10 h dark to maintain circadian rhythmicity. The use of zebrafish in our research is approved by the local animal welfare committee (DEC) of Leiden University (license 10,612) and reported according to the ARRIVE guidelines. In this study, adult zebrafish were used only for breeding of embryos. For the infection experiments, embryos were used before reaching the stage (5 dpf) from which free-feeding larvae are subject to regulations for animal experimentation. Fertilization was performed by natural spawning at the beginning of the light period and eggs were raised at 28.5 °C in egg water (60 µg/mL Instant Ocean sea salts and 0.0025% methylene blue).

#### Construction of Mtb ***blaC*** in Mmar

Mmar Δ*blaC* strain was created as described before using *S. thermophilus* CRISPR1-Cas9^39^. sgRNAs were designed to target the *blaC* gene and pTdTomato-L5 was electroporated into the knock-out strain to replace pCRISPRx-Sth1Cas9-L5^39^. To introduce Mtb *blaC* variants on the chromosome of Mmar, the Mtb *blaC* gene (Uniprot P9WKD3) was cloned into the integration vector pML1337, replacing *psmyc-gfpm2*+^62^. Mtb *blaC* is preceded by the 140 bp of the upstream flanking region and signal peptide of Mmar *blaC* (Fig. S1). Mutations S70A, I105F, G132S, G132N, D172N, D179N, or K234R were introduced into the Mtb *blaC* gene, and plasmids were electroporated into the Mmar Δ*blaC* pTdTomato-L5 strain, replacing pTdTomato-L5 in the MMAR_5512 locus (attB recombination site, position 4733000..4733042 of sequence NC_010612.1). Colonies were screened for resistance against kanamycin and sensitivity to streptomycin, and the constructs were confirmed by sequencing.

#### Analysis of Mtb blaC gene expression in Mmar

Mmar cultures were grown to OD_600_ = 0.6–1.0 before isolating the total RNA using the GeneJET RNA Purification Kit (Thermo Scientific). RT-qPCR primers were designed to target Mtb *blaC, sigA* (MMAR_2011), and *rrs* (MMAR_5519) (Table S1), and measurements were performed with 30 ng RNA per reaction using the Luna^®^ Universal One-Step RT-qPCR Kit (BioRad) and CFX Opus 96 Real-Time PCR System (BioRad). Cq values were determined with CFX Maestro Software (BioRad), and the relative expression (2^−ΔΔCq^) was calculated by comparing *blaC* to *sigA.* Melting curve analysis confirmed the formation of a single product (Fig. S7).

#### Antibiotic resistance and inhibitor sensitivity

Antibiotic resistance and inhibitor susceptibility was tested by adding 10 μL of Mmar Δ*blaC* pML1337-Mtb *blaC* liquid cultures with optical densities of 0.3, 0.03, 0.003, and 0.0003 on plates with various concentrations of ampicillin and inhibitors. Plates were incubated for 8 days at 30 °C before imaging.

#### Zebrafish embryo toxicity test

One day-old zebrafish embryos (30 hpf) were manually dechorionated using surgical forceps (Dumont #5), anesthetized using 0.02% aminobenzoic acid ethyl ester (tricaine, Sigma Aldrich), and injected with 1 nL of either PBS, ampicillin, clavulanate, sulbactam or avibactam in PBS. The solutions contained 10% phenol red (Sigma-Aldrich) to aid visualization of injections. The solutions were injected into the blood island of the embryos using glass microcapillary needles and survival rates were tracked for 4 days.

#### Zebrafish embryo infection

To allow for imaging of the mycobacteria in zebrafish larvae, the pTEC15 plasmid (Addgene plasmid # 30174)^63^ was electroporated into the Mmar Δ*blaC* pML1337-Mtb *blaC* strains and colonies were selected for both kanamycin and hygromycin resistance. One day-old zebrafish embryos (30 hpf) were dechorionated, anesthetized, and injected in the blood island with 1 nL of Mmar pTEC15, Mmar Δ*blaC* pML1337_Mtb *blaC* pTEC15, or Mmar Δ*blaC* pML1337_Mtb *blaC_K234R* pTEC15 (300 cfu in PBS). At ~ 24 h post-infection, embryos showing systemic infection were injected in the Duct of Cuvier with 1 nL of either PBS, ampicillin, avibactam, or a combination of ampicillin and avibactam.

#### Image quantification and statistical analysis

Zebrafish larvae were anesthetized and imaged 4 dpi using a Leica M205FA fluorescence stereomicroscope equipped with a Leica DFC 345FX camera and Leica Las X software. Fluorescence intensity was quantified using QuantiFish version 2.1.1^64^. Intensities were normalized by using the mean of the control group as 100%. Many of the distributions are highly skewed and consequently, non-parametric statistical analysis was required to analyze the data (Fig. S6). In addition, there is unequal variance between groups. Mood’s median test with Holm-Bonferroni post hoc test for multiple comparisons was employed to compare the treated groups to the control (Figs. 3b and S5e) or treated groups for BlaC WT and K234R (Fig. 4b)^65^.

### Supplementary Information

Supplementary Information 1.

Supplementary Information 2.

## Data Availability

Data is available as a Supplementary Information file.

## References

[1] World Health Organization. *Global Tuberculosis Report 2021*. (2021).

[2] Russell DG (2011). *Mycobacterium tuberculosis* and the intimate discourse of a chronic infection. Immunol. Rev..

[3] Wang F, Cassidy C, Sacchettini JC (2006). Crystal structure and activity studies of the *Mycobacterium tuberculosis* β-lactamase reveal its critical role in resistance to β-lactam antibiotics. Antimicrob. Agents Chemother..

[4] Abraham EP, Chain E (1940). An enzyme from bacteria able to destroy penicillin. Nature.

[5] Brown AG (1976). Naturally occurring β-lactamase inhibitors with antibacterial activity. J. Antibiot. (Tokyo).

[6] Reading C, Cole M (1977). Clavulanic acid: A β-lactamase-inhibiting β-lactam from *Streptomyces clavuligerus*. Antimicrob. Agents Chemother..

[7] World Health Organisation. *WHO Consolidated Guidelines on Tuberculosis: Module 4: Treatment—Drug-Resistant Tuberculosis Treatment, 2022 Update*. (2022). 36630546

[8] Donald PR (2001). Early bactericidal activity of amoxicillin in combination with clavulanic acid in patients with sputum smear-positive pulmonary tuberculosis. Scand. J. Infect. Dis..

[9] Payen MC (2012). Clinical use of the meropenem-clavulanate combination for extensively drug-resistant tuberculosis. Int. J. Tuberc. Lung Dis..

[10] Cohen KA (2016). Paradoxical hypersusceptibility of drug-resistant *Mycobacterium tuberculosis* to β-lactam antibiotics. EBioMedicine.

[11] Diacon AH (2016). β-Lactams against tuberculosis—New trick for an old dog?. N. Engl. J. Med..

[12] Drawz SM, Bonomo RA (2010). Three decades of β-lactamase inhibitors. Clin. Microbiol. Rev..

[13] Lee N, Yuen K-Y, Kumana CR (2003). Clinical role of β-lactam/β-lactamase inhibitor combinations. Drugs.

[14] Bonnefoy A (2004). *In vitro* activity of AVE1330A, an innovative broad-spectrum non-β-lactam β-lactamase inhibitor. J. Antimicrob. Chemother..

[15] Tassoni R, Blok A, Pannu NS, Ubbink M (2019). New conformations of acylation adducts of inhibitors of β-lactamase from *Mycobacterium tuberculosis*. Biochemistry.

[16] van Alen I (2021). The G132S mutation enhances the resistance of *Mycobacterium tuberculosis* β-lactamase against sulbactam. Biochemistry.

[17] Kurz SG (2013). Can inhibitor-resistant substitutions in the *Mycobacterium tuberculosis* β-lactamase BlaC lead to clavulanate resistance?: A biochemical rationale for the use of β-lactam-β-lactamase inhibitor combinations. Antimicrob. Agents Chemother..

[18] Egesborg P, Carlettini H, Volpato JP, Doucet N (2015). Combinatorial active-site variants confer sustained clavulanate resistance in BlaC β-lactamase from *Mycobacterium tuberculosis*. Protein Sci..

[19] Soroka D (2015). Hydrolysis of clavulanate by *Mycobacterium tuberculosis* β-lactamase BlaC harboring a canonical SDN motif. Antimicrob. Agents Chemother..

[20] Elings W (2021). Two β-lactamase variants with reduced clavulanic acid inhibition display different millisecond dynamics. Antimicrob. Agents Chemother..

[21] Feiler C (2013). Directed evolution of *Mycobacterium tuberculosis* β-lactamase reveals gatekeeper residue that regulates antibiotic resistance and catalytic efficiency. PLoS ONE.

[22] van Alen I (2023). Asp179 in the class A β-lactamase from *Mycobacterium tuberculosis* is a conserved yet not essential residue due to epistasis. FEBS J..

[23] Myllymäki H, Bäuerlein CA, Rämet M (2016). The zebrafish breathes new life into the study of tuberculosis. Front. Immunol..

[24] Cronan MR, Tobin DM (2014). Fit for consumption: Zebrafish as a model for tuberculosis. DMM Dis. Models Mech..

[25] Matty MA, Roca FJ, Cronan MR, Tobin DM (2015). Adventures within the speckled band: Heterogeneity, angiogenesis, and balanced inflammation in the tuberculous granuloma. Immunol. Rev..

[26] Varela M, Meijer AH (2022). A fresh look at mycobacterial pathogenicity with the zebrafish host model. Mol. Microbiol..

[27] Parikka M (2012). *Mycobacterium marinum* causes a latent infection that can be reactivated by gamma irradiation in adult zebrafish. PLoS Pathog..

[28] Stinear TP (2008). Insights from the complete genome sequence of *Mycobacterium marinum* on the evolution of *Mycobacterium tuberculosis*. Genome Res..

[29] Clay H (2007). Dichotomous role of the macrophage in early *Mycobacterium marinum* infection of the zebrafish. Cell Host Microbe.

[30] Swaim LE (2006). *Mycobacterium marinum* infection of adult zebrafish causes caseating granulomatous tuberculosis and is moderated by adaptive immunity. Infect. Immun..

[31] Van Der Sar AM (2004). *Mycobacterium marinum* strains can be divided into two distinct types based on genetic diversity and virulence. Infect. Immun..

[32] Meijer AH (2016). Protection and pathology in TB: Learning from the zebrafish model. Semin. Immunopathol..

[33] Yang CT (2012). Neutrophils exert protection in the early tuberculous granuloma by oxidative killing of mycobacteria phagocytosed from infected macrophages. Cell Host Microbe.

[34] Ramakrishnan L (2012). Revisiting the role of the granuloma in tuberculosis. Nat. Rev. Immunol..

[35] Davis JM, Ramakrishnan L (2009). The role of the granuloma in expansion and dissemination of early tuberculous infection. Cell.

[36] Matty MA (2019). Potentiation of P2RX7 as a host-directed strategy for control of mycobacterial infection. Elife.

[37] Boland R (2023). Repurposing tamoxifen as potential host-directed therapeutic for tuberculosis. MBio.

[38] Bhandari M (2023). Subcellular localization and therapeutic efficacy of polymeric micellar nanoparticles encapsulating bedaquiline for tuberculosis treatment in zebrafish. Biomater. Sci..

[39] Meijers AS (2020). Efficient genome editing in pathogenic mycobacteria using *Streptococcus thermophilus* CRISPR1-Cas9. Tuberculosis.

[40] Ambler RP (1991). A standard numbering scheme for the class A β-lactamases. Biochem. J..

[41] Soroka D (2017). Inhibition of β-lactamases of mycobacteria by avibactam and clavulanate. J. Antimicrob. Chemother..

[42] Papp-Wallace KM, Winkler ML, Taracila MA, Bonomo RA (2015). Variants of β-lactamase KPC-2 that are resistant to inhibition by avibactam. Antimicrob. Agents Chemother..

[43] Chikunova A, Ubbink M (2022). The roles of highly conserved, non-catalytic residues in class A β-lactamases. Protein Sci..

[44] Mehta AC, Hart-Davies S, Paynet J, Lacey RW (1994). Stability of amoxycillin and potassium clavulanate in co-amoxiclav oral suspension. J. Clin. Pharm. Ther..

[45] Peace N, Olubukola O, Moshood A (2012). Stability of reconstituted amoxicillin clavulanate potassium under simulated in-home storage conditions. J. Appl. Pharm. Sci..

[46] Zimmermann W, Rosselet A (1977). Function of the outer membrane of *Escherichia coli* as a permeability barrier to beta-lactam antibiotics. Antimicrob. Agents Chemother..

[47] Jarlier V, Nikaido H (1990). Permeability barrier to hydrophilic solutes in *Mycobacterium chelonei*. J. Bacteriol..

[48] Parish T, Brown AC (2008). Mycobacteria Protocols.

[49] Camacho LR (2001). Analysis of the phthiocerol dimycocerosate locus of *Mycobacterium tuberculosis*. Evidence that this lipid is involved in the cell wall permeability barrier. J. Biol. Chem..

[50] Ortalo-Magné A (1996). Identification of the surface-exposed lipids on the cell envelopes of *Mycobacterium tuberculosis* and other mycobacterial species. J. Bacteriol..

[51] Rennekamp AJ, Peterson RT (2015). 15 years of zebrafish chemical screening. Curr. Opin. Chem. Biol..

[52] Bootorabi F (2017). Zebrafish as a model organism for the development of drugs for skin cancer. Int. J. Mol. Sci..

[53] Cassar S (2020). Use of zebrafish in drug discovery toxicology. Chem. Res. Toxicol..

[54] Gustafson AL (2012). Inter-laboratory assessment of a harmonized zebrafish developmental toxicology assay—Progress report on phase I. Reprod. Toxicol..

[55] Ordas A (2015). Testing tuberculosis drug efficacy in a zebrafish high-throughput translational medicine screen. Antimicrob. Agents Chemother..

[56] Habjan E (2021). An anti-tuberculosis compound screen using a zebrafish infection model identifies an aspartyl-tRNA synthetase inhibitor. Dis. ModelS Mech..

[57] Dubée V (2014). β-Lactamase inhibition by avibactam in *Mycobacterium abscessus*. J. Antimicrob. Chemother..

[58] Lefebvre AL (2017). Inhibition of the β-lactamase Bla_Mab_ by avibactam improves the *in vitro* and *in vivo* efficacy of imipenem against *Mycobacterium abscessus*. Antimicrob. Agents Chemother..

[59] Jabila Mary TR (2021). β-lactamase inhibitory potential of kalafungin from marine *Streptomyces* in *Staphylococcus aureus* infected zebrafish. Microbiol. Res..

[60] Ramakrishnan L, Falkow S (1994). *Mycobacterium marinum* persists in cultured mammalian cells in a temperature-restricted fashion. Infect. Immun..

[61] Smeets LC (2006). Functional characterization of the competence protein DprA/Smf in *Escherichia coli*. FEMS Microbiol. Lett..

[62] Huff J, Czyz A, Landick R, Niederweis M (2010). Taking phage integration to the next level as a genetic tool for mycobacteria. Gene.

[63] Takaki K, Davis JM, Winglee K, Ramakrishnan L (2013). Evaluation of the pathogenesis and treatment of *Mycobacterium marinum* infection in zebrafish. Nat. Protoc..

[64] Stirling DR (2020). Analysis tools to quantify dissemination of pathology in zebrafish larvae. Sci. Rep..

[65] Mood AM (1954). On the asymptotic efficiency of certain nonparametric two-sample tests. Ann. Math. Stat..

