# Supplementary material for: Mycobacterium tuberculosis β-lactamase variant reduces sensitivity to ampicillin/avibactam in a zebrafish-Mycobacterium marinum model of tuberculosis
Source: Sci Rep. 2023 Sep 16;13:15406. doi: 10.1038/s41598-023-42152-8 (PMC10505137; doi:10.1038/s41598-023-42152-8)
Supplement: Supplementary file 1 — Supplementary Information 1. [file 41598_2023_42152_MOESM1_ESM.pdf]

Supplementary Information to

Mycobacterium tuberculosis β-lactamase variant reduces sensitivity to ampicillin/avibactam in a zebrafish-*Mycobacterium marinum* model of tuberculosis

Ilona van Alen[1], Mayra A. Aguirre García[2], Janneke J. Maaskant[3], Coenraad P. Kuijl[3], Wilbert Bitter[3,4], Annemarie H. Meijer[2], Marcellus Ubbink[1,*]

[1] Leiden Institute of Chemistry, Leiden University, Einsteinweg 55, 2333CC Leiden, The Netherlands

[2] Institute of Biology Leiden, Leiden University, Einsteinweg 55, 2333CC Leiden, The Netherlands

[3] Department of Medical Microbiology and Infection Control, Amsterdam UMC, location VUmc, De Boelelaan 1108, 1081 HZ, Amsterdam, The Netherlands

[4] Section of Molecular Microbiology, Amsterdam Institute of Molecular and Life Sciences, Vrije Universiteit Amsterdam, De Boelelaan 1108, 1081 HZ, Amsterdam, The Netherlands

* Corresponding author: E-mail: m.ubbink@chem.leidenuniv.nl; phone: +31 71 5274628

Table S1. Primer pairs used for RT-qPCR. Technical replicates were performed using a different primer pairs per reaction.

Primer pair	Forward primer	Reverse primer
**Mtb *blaC* - 1**	CCACCGCAGCAATTGAATATCGTG	TCAGTTTATCCAGATGGGTCAGCG
**Mtb *blaC* - 2**	GTTATCTGCGTAGCCTGGGTGATAC	TGCATTACCCAGAACCAGCTGC
***sigA* (MMAR_2011) - 1**	AACAACAGGTCAAGCCGACGAC	ATCGTCAGCGGTGTCCAGATTG
***sigA* (MMAR_2011) - 2**	TCTCTACGCGACACAGCTGATG	GTTGGCTTCCAGCAGGTGGTTT
***rrs* (MMAR_5519) - 1**	TACGGGCAGACTAGAGTACTGCAG	CTTTCGCTCCTCAGCGTCAGTTAC
***rrs* (MMAR_5519) - 2**	GCGCAACCCTTGTCTCATGTTG	TGTACCGGCCATTGTAGCATGTG

A

GTCATGGGCAAATACCTTAACCGGGCAGGTGCGGCGCCCTACGACCGGCCGTCGTAGAGCGCCGCGGCGGGGCCGCCCCACGC
CGCAGCGCCGGGGAAGCGACACGCTGAAACCGGGATCGTGGGACACTCGTTCGCGATATGCGTCCCTCAAACCCGCGGTCGGC
GGTAAACCGGCGTCAGTTGCTAGCGGCGATGGCTGCGTTGCTCCCGCTTTCGGCATGCGCCAAAGCGGCCAGTGATCAACACA
TGGCCTCGACGATGGCGGTGCCCAGCCCGGATCTGGCAGATCGTTTTGCAGAACTGGAACGTCGTTATGATGCACGTCTGGGT
GTTTATGTTCCGGCAACCGGCACCACCGCAGCAATTGAATATCGTGCAGATGAACGTTTTGCATTTTGCAGCACCTTTAAAGC
ACCGCTGGTTGCAGCCGTTCTGCATCAGAATCCGCTGACCCATCTGGATAAACTGATTACCTATACCAGTGATGATATCCGTA
GCATTAGTCCGGTTGCACAGCAGCATGTTCAGACCGGTATGACCATTGGTCAGCTGTGTGATGCAGCAATTCGTTATAGTGAT
GGCACCGCAGCCAATCTGCTGCTGGCCGATCTGGGTGGACCGGGTGGTGGTACAGCAGCCTTTACCGGTTATCTGCGTAGCCT
GGGTGATACCGTTAGCCGTCTGGATGCAGAAGAACCGGAACTGAATCGTGATCCGCCTGGTGATGAACGTGATACCACCACAC
CGCATGCCATTGCACTGGTTCTGCAGCAGCTGGTTCTGGGTAATGCACTGCCTCCGGATAAACGTGCACTGCTGACCGATTGG
ATGGCACGTAATACCACCGGTGCCAAACGTATTCGTGCAGGTTTTCCGGCAGATTGGAAAGTTATTGATAAAACCGGTACGGG
TGATTATGGTCGTGCAAATGATATTGCAGTTGTTTGGAGCCCGACCGGTGTTCCGTATGTTGTTGCAGTTATGAGCGATCGTG
CCGGTGGTGGCTATGATGCCGAACCGCGTGAAGCACTGCTGGCGGAAGCAGCAACCTGTGTTGCCGGTGTTCTGGCACTCGAG
TA

B

```
                                                      30         40
MRPSNPRSAV NRRQLLAAMA ALLPLSACAK AASDQHMAST MAVPSPDLAD RFAELERRYD

         50         60          70          80        90         100
ARLGVYVPAT GTTAAIEYRA DERFAFCSTF KAPLVAAVLH QNPLTHLDKL ITYTSDDIRS

      110        120         130         140            150        160
ISPVAQQHVQ TGMTIGQLCD AAIRYSDGTA ANLLLADLGG PGGGTAAFTG YLRSLGDTVS

          170        180         190         200         210       220
RLDAEEPELN RDPPGDERDT TTPHAIALVL QQLVLGNALP PDKRALLTDW MARNTTGAKR

          230        240         250        260          270       280
IRAGFPADWK VIDKTGTGDY GRANDIAVVW SPTGVPYVVA VMSDRAGGGY DAEPREALLA

          290
EAATCVAGVL ALE
```

Figure S1. DNA and amino acid sequences of Mtb BlaC in Mmar. (A) The Mtb *blaC* gene is preceded by 140 bp of the upstream flanking region of Mmar *blaC* (shaded) and the sequence coding for the Mmar *blaC* signal peptide (underlined, locus MMAR_3050). (B) Residues 28-291 are numbered according to the Ambler notation[1] (BlaC misses residue 58, 84, 85, 239, and 253 and has 6 additional residues: 145A, B, C, D, 269A, and B; this corresponds to residue numbers 43-307 of Mtb BlaC Uniprot entry P9WKD3-1. The last two residues (L and E) are remnant of cloning. Residues of the Mmar signal peptide are underlined (residues 0-46 of Uniprot entry B2HFS3).

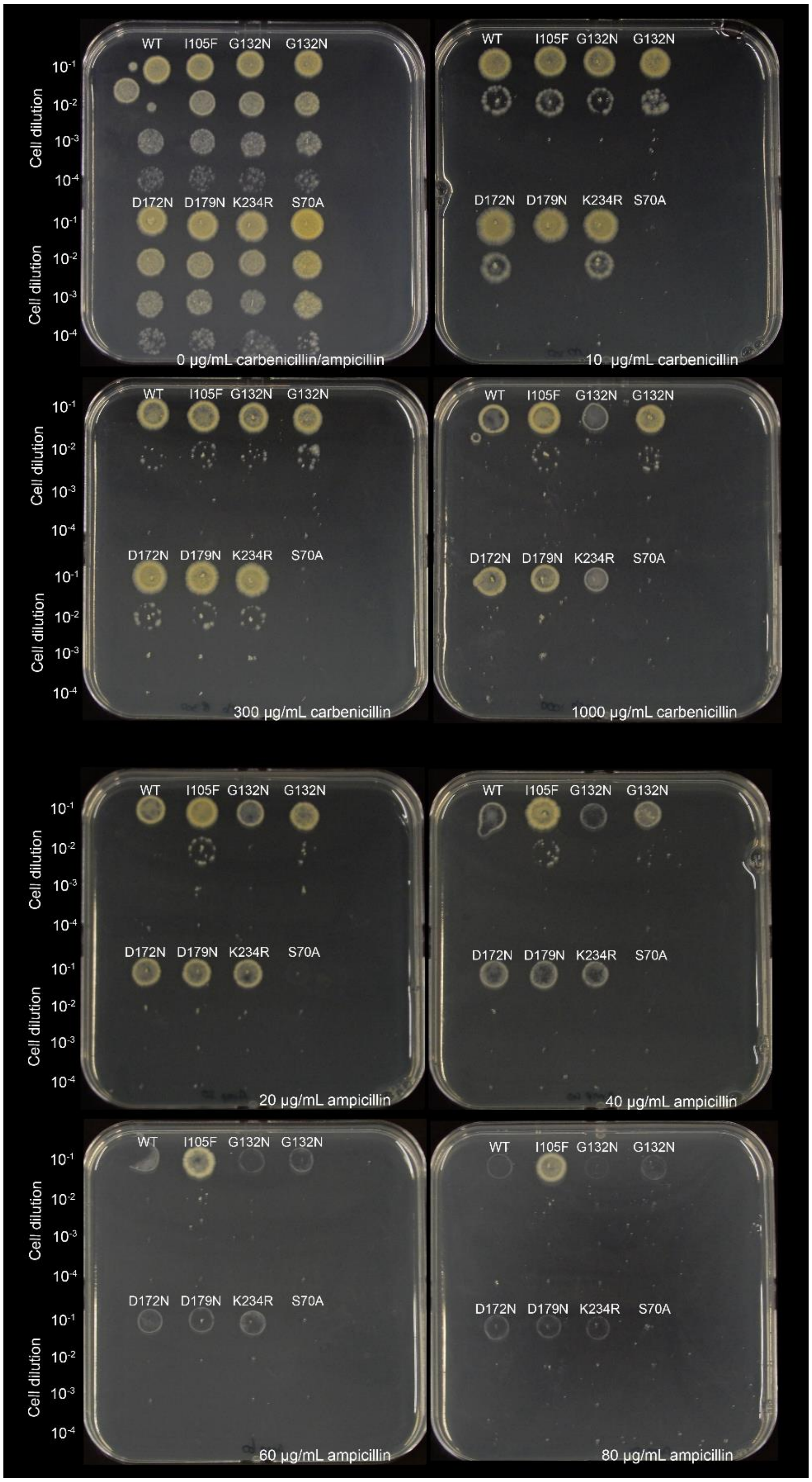

Figure S2. Activity of Mtb BlaC variants produced in Mmar. Cultures of Mmar chromosomally expressing wild type BlaC or variants S70A (negative control), I105F, G132N, G132S, D172N, D179N, and K234R were incubated for 8 days at 30 °C on plates containing indicated concentrations of carbenicillin and ampicillin.

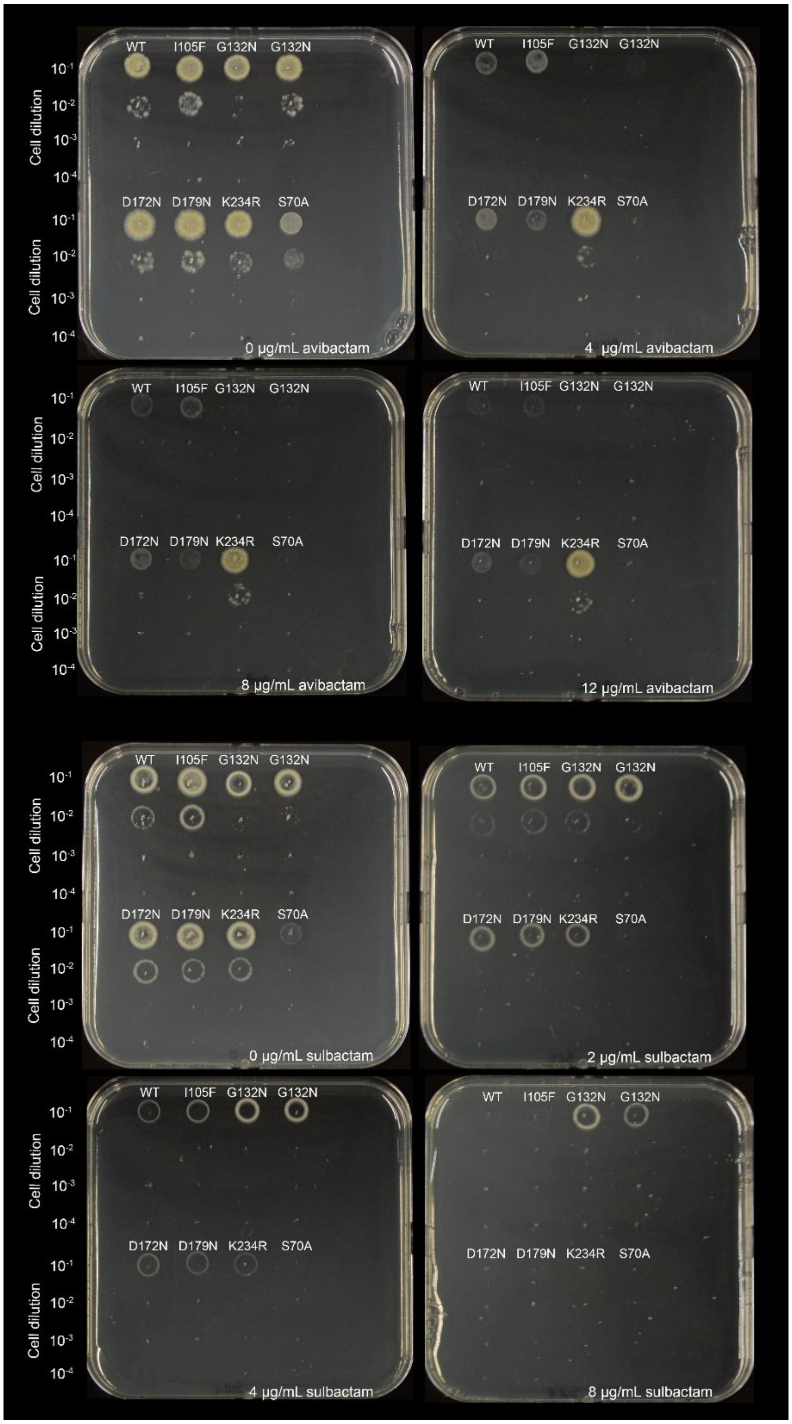

Figure S3. Activity of Mtb BlaC variants produced in Mmar. Cultures of Mmar chromosomally expressing wild type BlaC or variants S70A (negative control), I105F, G132N, G132S, D172N, D179N, and K234R were incubated for 8 days at 30 °C on plates containing indicated concentrations of avibactam and sulbactam in the presence of 15 µg mL^{-1} ampicillin.

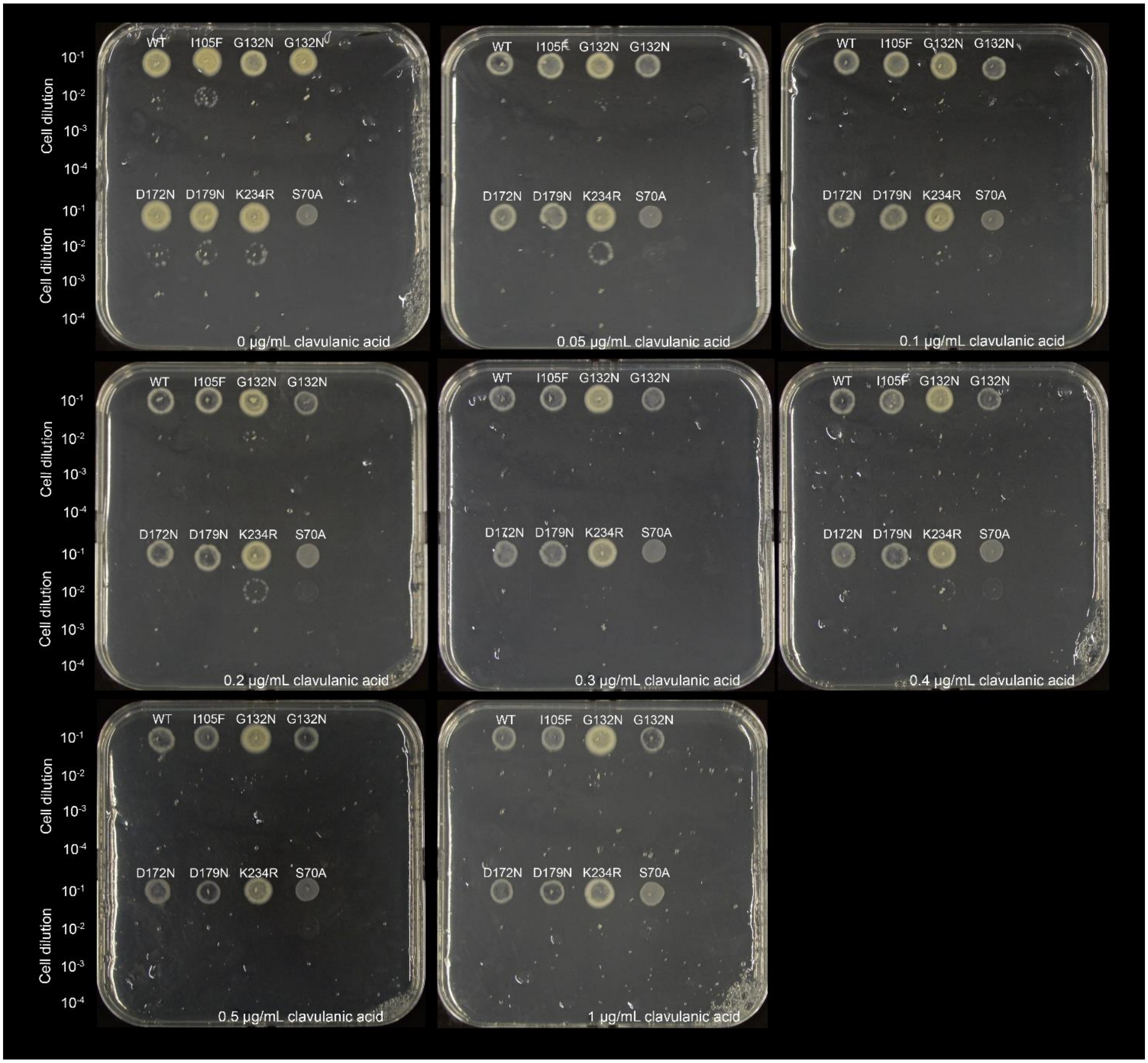

Figure S4. Activity of Mtb BlaC variants produced in Mmar. Cultures of Mmar chromosomally expressing wild type BlaC or variants S70A (negative control), I105F, G132N, G132S, D172N, D179N, and K234R were incubated for 8 days at 30 °C on plates containing indicated concentrations of clavulanic acid in the presence of 15 µg mL^{-1} ampicillin.

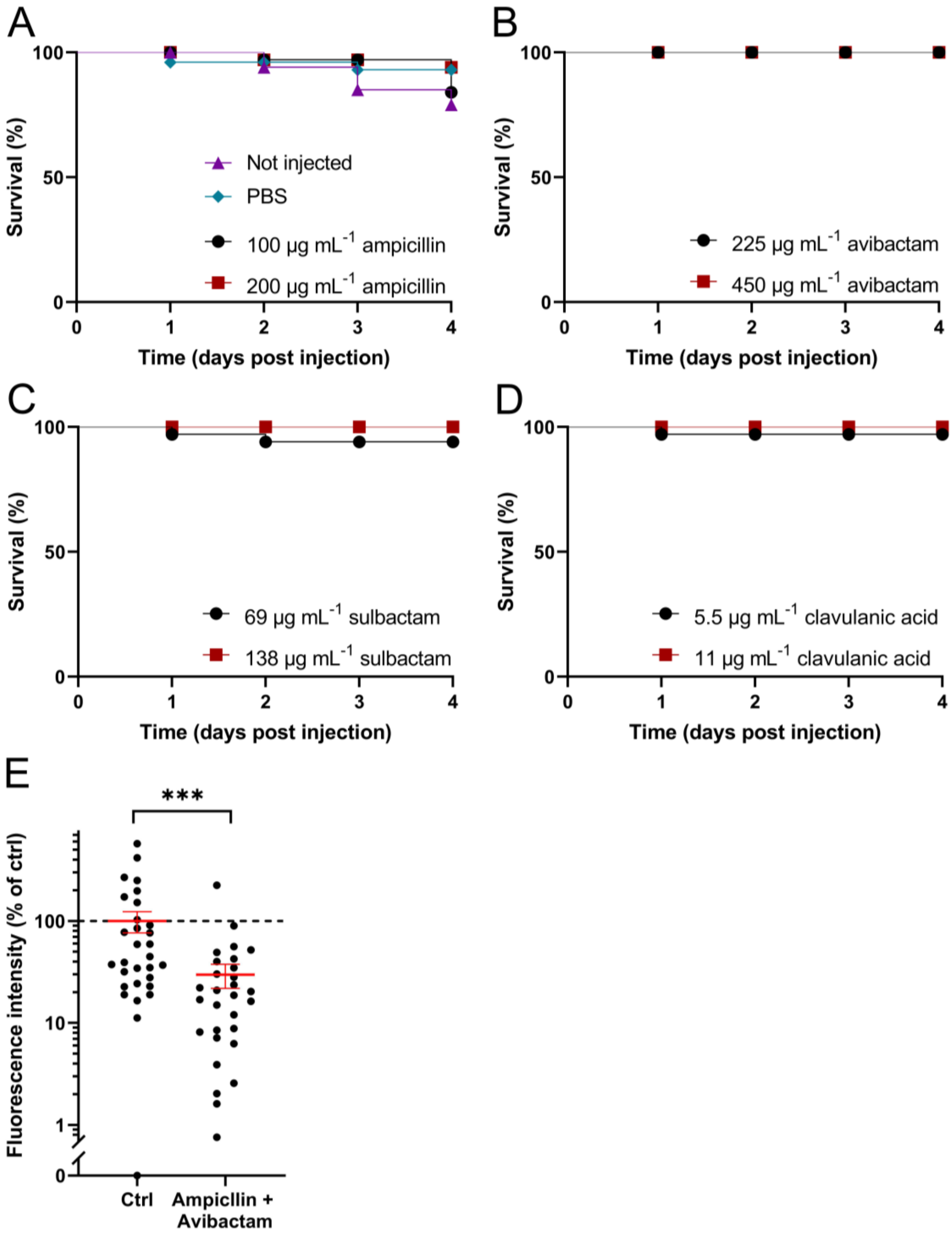

Figure S5. Effect of treatment by injection with ampicillin and/or inhibitors on zebrafish embryos. (A-D) Survival rates of embryos (A) not injected (n=34) or injected at 1dpf with 1 nL of either PBS (n=28), or ampicillin in PBS (29 mg mL^{-1}, n=32 or 58 mg mL^{-1} n=33), (B) avibactam in PBS (65 mg mL^{-1}, n=31 or 131 mg mL^{-1}, n=29), (C) sulbactam in PBS (20 mg mL^{-1}, n=35 or 40 mg mL^{-1}, n=23), (D) clavulanic acid in PBS (1.6 mg

mL^{-1} or 3.2 mg mL^{-1}, both n=31). The injected concentrations were calculated to reach the desired concentrations of 100 or 200 µg mL^{-1} ampicillin, 5.5 or 11 µg mL^{-1} clavulanic acid, 69 or 138 µg mL^{-1} sulbactam, or 225 or 450 µg mL^{-1} avibactam in the embryo. (E) Bacterial load of wild type Mmar represented by fluorescence intensity after being given the indicated treatment 1dpi. Larvae were injected with 1 nL of 29 mg mL^{-1} ampicillin and 22 mg mL^{-1} avibactam in PBS (estimated concentration 100 µg mL^{-1} ampicillin and 75 µg mL^{-1} avibactam in the embryo, n=29) or PBS only (ctrl, n=30) at 4 dpi. Mood's median test was used to compare the treatment group with the control: *** = p <0.001.

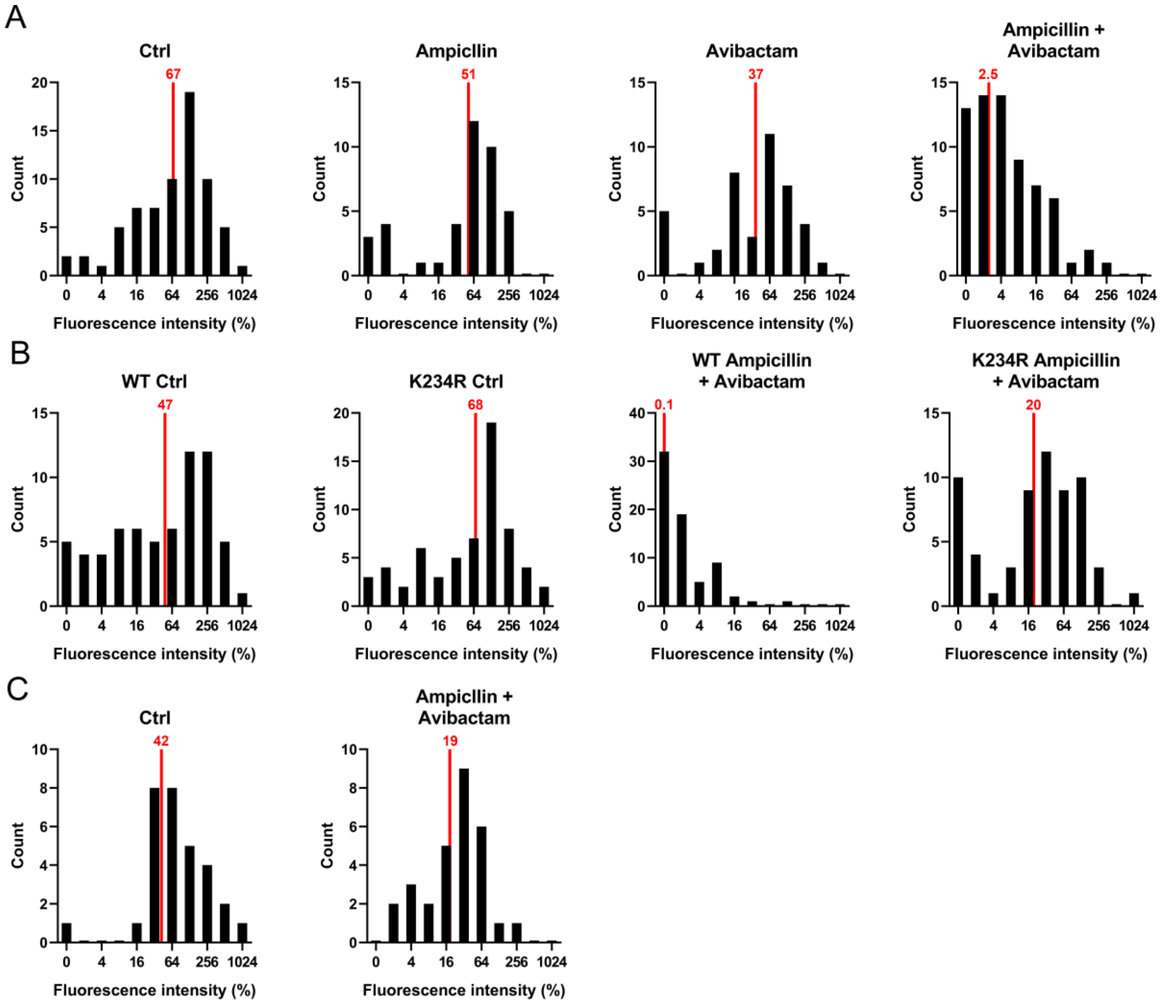

Figure S6. Distribution of fluorescence intensity for the Mmar infection datasets. (A) Number of larvae infected with Mmar producing wild type Mtb BlaC after been given the indicated treatment 1dpi (Figure 2b). Larvae were injected with 1 nL of 29 mg mL^{-1} ampicillin in PBS (estimated concentration 100 µg mL^{-1} in the embryo, n=39), 65 mg mL^{-1} avibactam in PBS (225 µg mL^{-1}, n=43), ampicillin and avibactam in PBS (same concentrations, n=67) or PBS only (ctrl, n=69). Data for the groups treated with both ampicillin and avibactam or the control were accumulated in three, and the groups treated with only ampicillin or avibactam in two independent experiments. (B) Number of larvae infected with Mmar producing either wild type or K234R Mtb BlaC after been given the indicated treatment 1dpi (Figure 2c). Larvae were injected with 1 nL of 29 mg mL^{-1} ampicillin and 22 mg mL^{-1} avibactam in PBS (estimated concentration 100 µg mL^{-1} ampicillin and 75 µg mL^{-1} avibactam in the embryo, n=69 for WT and n=62 for K234R) or PBS only (ctrl, n=66 for WT and n=63 for K234R). Data were accumulated in three independent experiments. (C) Number of larvae infected with wild type Mmar after being given the indicated treatment 1dpi (Figure S5e). Larvae were injected with 1 nL of 29 mg mL^{-1} ampicillin and 22 mg mL^{-1} avibactam in PBS (estimated

concentration 100 µg mL^{-1} ampicillin and 75 µg mL^{-1} avibactam in the embryo, n=29) or PBS only (ctrl, n=30) at 4 dpi. Data were normalized by setting the mean of the control to 100%. The bin ranges are 0%, 0.01% - 2%, 2.01% - 4%, 4.01% - 8%, 8.01% - 16%, etc., with the maximum values as labels below the x-axis. Medians are shown in red.

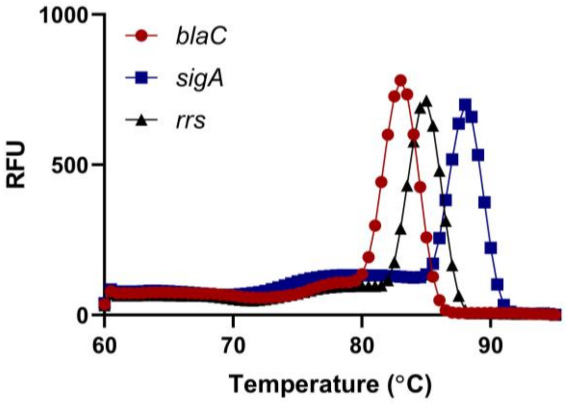

Figure S7. Melting curves of RT-qPCR amplicons of Mtb *blaC, sigA*, and *rrs* expressed in Mmar (Figure 1a).

References

1. Ambler, R. P. *et al.* A standard numbering scheme for the class A β-lactamases. *Biochemical Journal* **276**, 269–270 (1991).
